# Group rehabilitation for adults with acquired neurological disorders: A systematic review of mono‐ and interdisciplinary interventions in physical and speech‐language therapy

**DOI:** 10.1002/pmrj.70006

**Published:** 2025-11-11

**Authors:** Nina Greiner, Norina Lauer, Valentin Schedel, Andrea Pfingsten

**Affiliations:** ^1^ Regensburg Center of Health Sciences and Technology (RCHST)/Faculty of Social and Health Care Sciences OTH Regensburg Regensburg Germany

## Abstract

**Background:**

Group treatments and interdisciplinary collaboration are recommended in evidence‐based guidelines for neurorehabilitation, including physical and speech‐language therapy. Evidence suggests that activating overlapping neural networks for upper extremity motor control and speech‐language processing produces synergistic effects during therapy. This systematic review aims to overview and appraise the efficacy of group treatments in traditional rehabilitation and telerehabilitation. In addition to summarizing evidence on monodisciplinary approaches in physical and speech‐language therapy, it seeks data on integrative approaches involving one or both disciplines to inform further interdisciplinary collaboration.

**Methods:**

The review was registered with PROSPERO (CRD42021288012) and followed the Preferred Reporting Items for Systematic Reviews and Meta‐Analyses (PRISMA) guidelines. Systematic searches were conducted in PubMed, CINAHL, and the Cochrane Library. Two reviewers independently screened studies, extracted data, and assessed quality using AMSTAR 2, the Physiotherapy Evidence Database (PEDro) scale, or the Joanna Briggs Institute (JBI) Checklist, as appropriate. The evidence was summarized in a systematic narrative synthesis and its certainty rated based on the Grading of Recommendations Assessment, Development, and Evaluation (GRADE) approach.

**Results:**

A total of 29 studies were included: 16 on speech‐language therapy (861 participants) and 13 on physical therapy (1757 participants). No studies addressed interdisciplinary group interventions, and only two evaluated group telerehabilitation. Outcome domains and measures varied across studies and the certainty of evidence was predominantly low. However, moderate‐certainty evidence supports that group speech‐language therapy improves quality of life, communication, and language in stroke survivors, especially when interventions emphasize verbal production in communicative settings with multimodal materials and cueing. In physical therapy, circuit class training may be more effective than other group approaches for enhancing quality of life and mobility.

**Conclusion:**

Group treatments in neurorehabilitation show some benefits, but further research is needed – especially regarding interdisciplinary approaches and telerehabilitation.

## INTRODUCTION

Group treatments are a key component of rehabilitation for acquired neurological disorders and are recommended in evidence‐based guidelines for neurorehabilitation, including speech‐language therapy (SLT) and physical therapy (PT).^1^, ^2^, ^3^, ^4^, ^5^, ^6^ Evidence suggests that group therapy improves outcomes such as language, communication, and quality of life in aphasia;^7^, ^8^, ^9^ intelligibility in dysarthria;^10^ balance, mobility, and gait speed in individuals with hemiparesis,^11^, ^12^, ^13^ and symptoms of post‐stroke depression.^14^ Recent advancements include group telerehabilitation – which refers to delivering interventions via information and communication technologies^15^ – an approach that shows promise despite heterogeneous results.^16^, ^17^, ^18^, ^19^ Interdisciplinary treatment is another essential recommendation as it appears especially applicable to the complex symptoms of neurologic patients^20^, ^21^, ^22^, ^23^ within the framework of the biopsychosocial model^24^ and the International Classification of Functioning, Disability and Health (ICF)^25^ (see Supplement A for all acronyms used in the text). This is supported by evidence of better patient outcomes with coordinated interdisciplinary rehabilitation.^22^, ^26^, ^27^ An integrated co‐treatment scheme involving SLT and PT appears to be particularly promising for group neurorehabilitation. Upper extremity (UE) motor status and language performance were shown to be associated in individuals with aphasia after brain damage,^28^, ^29^ likely due to the significant overlap between neural networks for UE motor control and those involved in speech and language processing.^30^, ^31^, ^32^, ^33^ Patients appear to benefit from the activation of overlapping neural networks, as evidenced by a positive correlation between motor and language gains in the rehabilitation process,^34^, ^35^, ^36^, ^37^ and robot‐assisted upper limb training improving both motor function and speech‐language performance.^38^, ^39^ The synergistic effect is probably further supported by the positive impact of physical exercise at the cellular level with increased neuroplastic change,^40^, ^41^ leading to functional improvements not only in the motor but also in the cognitive and linguistic domain.^34^, ^39^, ^42^, ^43^ An interdisciplinary group rehabilitation also allows for addressing important principles of neuroplasticity and brain reorganization that have been formulated^44^ and extended^45^ to guide treatment design: Key aspects include massed, task‐specific, goal‐oriented, variable and multimodal practice as well as feedback, action observation, and social interaction. Indeed, a pilot group telerehabilitation combining PT and SLT for patients with stroke with hemiparesis and aphasia and/or dysarthria indicated benefits.^46^, ^47^

Against this background, we conducted this systematic review to gather evidence on group rehabilitation for acquired neurological disorders. The aim was to provide an overview and appraise the efficacy of monodisciplinary group treatments in PT and SLT as well as interdisciplinary approaches combining PT and SLT with each other or with other disciplines. Further objectives were to identify shared concepts that could serve as common ground for promoting interdisciplinary PT/SLT approaches, as well as to describe the extent to which group treatments are already investigated and implemented in a telerehabilitation setting.

## METHODS

The review was preregistered (PROSPERO‐ID CRD42021288012) and followed the updated Preferred Reporting Items for Systematic Reviews and Meta‐Analyses (PRISMA) statement^48^ and its extension for searching.^49^

### Eligibility criteria

We conducted a systematic search for studies of adults with acquired neurological disorders. Publications dealing with neurodegenerative disorders, neurotoxic damage, or cerebral neoplasms were excluded. Eligible trials had to incorporate at least four sessions of monodisciplinary group‐based interventions from PT or SLT or an interdisciplinary treatment. The involved speech‐language pathologist or physical therapist had to contribute at least 50% of the therapeutic content. Group sessions were required to involve two patients at minimum and one or more facilitators. Face‐to‐face as well as screen‐to‐screen interventions were encompassed in our analysis, with the requirement that they applied outcome measures of functions, activities, participation, or quality of life. We included publications in English and German due to the authors' core language proficiency. The considered trials were feasibility or intervention studies (randomized controlled trials [RCTs] and non‐RCTs). Trials without control group, qualitative studies, and single case studies were excluded. In addition to primary studies, systematic reviews were incorporated to acknowledge the hierarchy of evidence and to leverage the work already done by other researchers. Systematic reviews were included only if all contained primary studies met the eligibility criteria. Otherwise, they were excluded as a whole, or, if applicable, only eligible primary publications were extracted from the review and brought into the screening process. Selected systematic reviews were checked for overlapping primary publications. When there was >50% overlap – compared to other included reviews – the review was excluded. Accordingly, primary studies identified in the search were excluded if they were part of an included systematic review.

### Information sources and search strategy

The review group consisted of a researcher and a research associate each from PT and SLT. The interdisciplinary team developed and discussed the search strategy using the Population, Intervention, Comparison, Outcomes (PICO) framework.^50^ It was optimized^51^ by adding PubMed filters (*therapy – sensitive/broad*,^52^ *systematic reviews filter* ^53^) and tested in a pilot search. The final electronic search was performed in the databases PubMed, CINAHL, and Cochrane Library in December 2022 (the search string can be found in Supplement B). Additional publications were identified by handsearching relevant journals and checking the reference lists of selected publications.

### Data management, extraction, and synthesis

We used Citavi and Zotero for reference management, and Rayyan^54^ for screening and handling the decisions. In the first screening stage, two research associates (N.G. and N.M.) with a background in PT and SLT, respectively, independently screened the titles and abstracts of the retrieved publications. Due to a staff change, all subsequent steps in the review, beginning with the full text screening in the second stage, were carried out by N.G. and V.S. The two research associates resolved screening conflicts through discussion, with the other group members (N.L. and A.P.) available for mediation if needed. Data extraction was done separately for the two therapeutic disciplines using a form that was created prior to the search. A systematic narrative approach was applied for synthesizing the evidence.^55^ The results of the studies were summarized and categorized by outcomes and comparison group.

### Critical appraisal

Critical appraisal was done independently by two review authors (N.G. and V.S.), the results were then compared, and any disagreements resolved by discussion. The other two members of the review group (N.L. and A.P.) were available to mediate if necessary. Depending on the study design, different tools were used: AMSTAR 2^56^ for systematic reviews, the PEDro scale^57^ for RCTs, and the JBI‐Checklist for Quasi‐Experimental Studies^58^ for controlled clinical trials (CCT). For the RCTs and CCTs, we defined the risk of bias level as low (PEDro score 6–8^59^; JBI rating “yes” for more than half of the criteria), unclear (PEDro score 4–5; JBI rating “unclear” for more than half of the criteria), or high (PEDro score 0–3; JBI rating “no” for more than half of the criteria). Subsequently, the certainty of evidence was determined for each outcome following the Grading of Recommendations Assessment, Development, and Evaluation (GRADE) approach.^60^ Publication bias could not be assessed because the number of studies for each outcome in each comparison group was below the Cochrane recommendations for using funnel plots.^61^ If both primary studies and a systematic review contributed to an outcome within a comparison group, certainty of evidence was based on AMSTAR 2 unless at least three primary studies differed (for details see Supplement C).

## RESULTS

### Identification of studies

**FIGURE 1:**
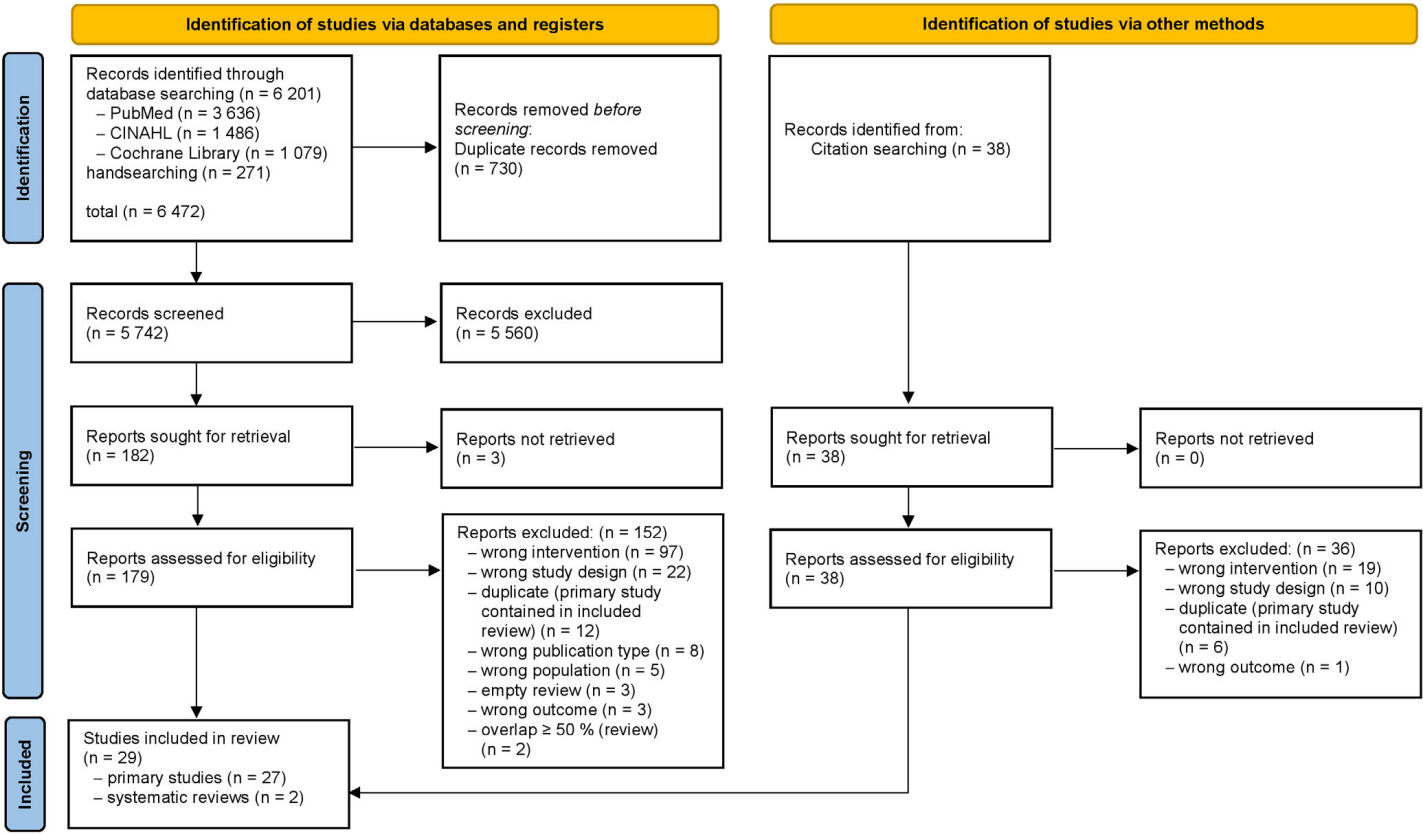
PRISMA flow diagram depicting the details of the searching and screening process.

The electronic database search identified 6201 publications. A further 271 studies were found by handsearching journals and 38 by checking reference lists of included publications. After the removal of duplicates, and the exclusion of 5742 articles based on title and abstract screening, 217 full text publications were retrieved and assessed for eligibility, resulting in the inclusion of 29 studies (Figure 1).

### Included studies

#### Study characteristics

Of 29 included studies, 16 addressed SLT group treatments (1 systematic review, 12 RCTs, 3 CCTs; 861 participants). The other 13 publications addressed group treatment in PT (1 systematic review, 9 RCTs, 3 CCTs; 1757 participants). Two of the 12 PT primary studies^62^, ^63^ are considered as a unit as they refer to the same study population and intervention and only report different outcomes. See Supplement D for the location of the studies, treatment doses, group sizes, and further details.

Overall, the predominant etiology was stroke, with only three studies conducted exclusively or partially on patients with traumatic brain injury (TBI).^64^, ^65^, ^66^ The mode of delivery was exclusively face‐to‐face in PT interventions and predominantly face‐to‐face in SLT interventions, with only two studies investigating telerehabilitation using a non‐^67^ or semi‐immersive^68^ virtual reality (VR) approach.^69^

None of the reviewed studies fully implemented an interdisciplinary approach in the sense of creating an integrated treatment, rather than merely juxtaposing the disciplines.^23^ Only three studies included some interdisciplinary elements by combining SLT interventions with psychological treatment approaches^64^, ^70^ or with art and drama therapy.^7^ Therefore, we decided not to create a separate category for interdisciplinary interventions. Instead, these studies are subsumed under the label of SLT.

#### Critical appraisal

The results of the AMSTAR 2 tool^56^ applied to rate the quality of the two systematic reviews can be found in Table 1. Tables 2 and 3 contain the risk of bias (RoB) determination for the included RCTs (PEDro scale^57^) and CCTs (JBI‐Checklist for Quasi‐Experimental Studies^58^), respectively. The rating results for certainty of evidence according to GRADE are summarized in Table 4.

**TABLE 1:** Critical appraisal of included systematic reviews according to AMSTAR 2.

AMSTAR 2 item	Discipline/study	
	PT English et al.^74^	SLT Zhang et al.^80^
PICO included in research question/inclusion criteria	Yes	Yes
Methods/protocol established prior to review	Yes	No
Selection of study design explained	No	No
Comprehensive search strategy used	Yes	No
Study selection performed in duplicate	Yes	Yes
Data extraction performed in duplicate	Yes	Yes
List/justification of excluded studies provided	Yes	No
Included studies described in detail	Partial yes	Partial yes
Satisfactory technique for RoB assessment used	Yes	Partial yes
Sources of funding reported for includes studies	No	No
Appropriate methods for meta‐analysis used	Yes	No
Impact of RoB assessed	Yes	No
RoB in interpretation/discussion accounted for	Yes	No
Heterogeneity explained/discussed	Yes	Yes
Publication bias investigated	No	No
Conflict of interest reported	No	Yes
Overall quality	Low	Critically low

Abbreviations: PT, physical therapy; RoB, risk of bias; SLT, speech‐language therapy.

**TABLE 2:** Critical appraisal of RCTs according to PEDro.

Discipline/study		PEDro criterion											
		Eligibility criteria^a^	Allocation		Similar at baseline	Blinding			Dropout at most 15%	Intention to treat	Between group comparison	Point measures/measures of variability	Score
			Random	Concealed		Subjects	Therapists	Assessors					
PT	Doussoulin et al.^62^	Yes	Yes	Yes	Yes	No	No	Yes	Yes	Yes	Yes	Yes	8
PT	Doussoulin et al.^63^	Yes	Yes	Yes	Yes	No	No	Yes	Yes	Yes	Yes	Yes	8
PT	Kleffelgaard et al.^65^	Yes	Yes	Yes	Yes	No	No	Yes	Yes	Yes	Yes	Yes	8
PT	Malagoni et al.^77^	Yes	Yes	Yes	Yes	No	No	No	Yes	Yes	Yes	Yes	7
PT	Martins et al.^72^	Yes	Yes	Yes	No	No	No	Yes	No	Yes	Yes	Yes	7
PT	Noh et al.^78^	Yes	Yes	No	Yes	Yes	No	Yes	No	No	Yes	Yes	5
PT	Renner et al.^12^	Yes	Yes	Yes	No	No	No	No	Yes	Yes	Yes	Yes	7
PT	Stein et al.^79^	Yes	Yes	Yes	No	No	Yes	Yes	Yes	Yes	Yes	Yes	8
PT	Thieme et al.^76^	Yes	Yes	Yes	Yes	No	No	Yes	No	Yes	Yes	Yes	7
SLT	Agrela et al.^64^	Yes	Yes	No	Yes	No	No	Yes	Yes	Yes	Yes	Yes	7
SLT	Elman & Bernstein‐Ellis^7^	Yes	Yes	No	Yes	No	No	No	No	No	Yes	Yes	4
SLT	Giachero et al.^68^	Yes	Yes	Yes	No	No	No	Yes	No	No	Yes	Yes	5
SLT	Kristensson et al.^89^	Yes	Yes	Yes	Yes	No	No	Yes	Yes	No	Yes	Yes	7
SLT	Küst et al.^82^	Yes	Yes	Yes	No	No	No	No	No	No	Yes	No	3
SLT	Marshall et al.^67^	Yes	Yes	Yes	Yes	No	No	No	Yes	Yes	Yes	Yes	7
SLT	Mohr et al.^66^	Yes	Yes	Yes	Yes	No	No	Yes	Yes	Yes	Yes	Yes	8
SLT	Nenert et al.^84^	Yes	Yes	No	Yes	No	No	No	Yes	No	Yes	Yes	5
SLT	Rose et al.^87^	Yes	Yes	Yes	No	No	No	Yes	Yes	No	Yes	Yes	6
SLT	Stahl et al.^86^	Yes	Yes	Yes	Yes	No	No	Yes	Yes	Yes	Yes	Yes	8
SLT	Vuksanović et al.^85^	Yes	Yes	Yes	Yes	No	No	Yes	Yes	Yes	Yes	Yes	8
SLT	Wertz et al.^88^	Yes	Yes	No	Yes	Yes	No	Yes	No	No	Yes	No	4

Abbreviations: PEDro, Physiotherapy Evidence Database; PT, physical therapy; RCT, randomized controlled trial; SLT, speech‐language therapy.

^a^ This criterion is not part of the total PEDro score.

**TABLE 3:** Critical appraisal of CCTs according to the JBI checklist.

Discipline/study		JBI criterion								
		Clear distinction of cause/effect	Participants similar	Similar treatment/care	Control group	Multiple measurements pre/post	Follow‐up complete/differences analyzed	Outcomes measured		Statistical analysis appropriate
								in the same way	in a reliable way	
PT	English et al.^71^	Yes	No	Yes	Yes	No	No	Yes	Yes	Yes
PT	Stibrant Sunnerhagen^73^	Yes	Unclear	No	Yes	No	N/a	Yes	Yes	Yes
PT	Dickstein et al.^75^	Yes	Unclear	Unclear	Yes	No	Unclear	Yes	Yes	Yes
SLT	Barthel et al.^81^	Yes	Yes	Unclear	Yes	No	Yes	Yes	Yes	Yes
SLT	Meinzer et al.^83^	Yes	Yes	Yes	Yes	No	No	Yes	Yes	Yes
SLT	Marshall et al.^70^	Yes	No	Yes	Yes	Yes	Yes	Yes	Yes	No

Abbreviations: CCT, controlled clinical trial; JBI, Joanna Briggs Institute; PT, physical therapy; SLT, speech‐language therapy.

**TABLE 4:** Summary of findings.

Stroke					
Comparison	Outcome	Discipline	Number/types of studies (participants)	Certainty of evidence (GRADE)^a^	Comment
Group and no intervention	Quality of life	SLT	1 RCT (216 Ps)	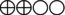 low	Downgraded to low because Ps were not similar at baseline and evidence comes from only one study.
	Communication	SLT	1 SR (1 study with 28 Ps, not part of meta‐analysis), 4 RCTs (323 Ps)	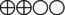 low	Downgraded to low because of high RoB of SR, unclear RoB of one RCT, and partially inconsistent findings and heterogenous outcome measurement instruments.
	Mobility	PT	1 SR (269 Ps)	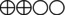 low	Downgraded because of low‐quality SR.
	Language	SLT	1 SR (1 study with 28 Ps, not part of meta‐analysis), 3 RCTs (303 Ps)	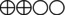 low	Downgraded to low because of high RoB of SR, unclear RoB of one RCT, and partially inconsistent findings.
	Emotional well‐being	SLT	1 RCT (20 Ps)	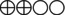 low	Downgraded to low because Ps were not similar at baseline and evidence comes from only one study with a small sample size.
Group and individual intervention	Quality of life	PT	1 RCT (12 Ps)	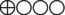 very low	Downgraded due to very low number of Ps in one single study.
	Communication	SLT	2 RCTs (95 Ps), 1 CCT (39 Ps)	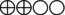 low	Downgraded to low because of high RoB of one RCT, unclear RoB of one RCT and partially inconsistent findings.
	Mobility	PT	4 RCTs (145 Ps), 1 CCT (78 Ps)	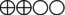 low	Downgraded to low because of inconsistency of effect and indirectness of surrogate outcome.
	Balance	PT	3 RCTs (175 Ps)	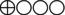 very low	Downgraded due to inconsistency and indirectness – control interventions not comparable.
	Language	SLT	1 SR (1 study with 20 Ps, not part of meta‐analysis), 2 RCTs (95 Ps), 1 CCT (39 Ps)	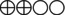 low	Downgraded to low because of high RoB of SR, high RoB of one RCT, unclear RoB of another RCT and small inconsistencies in findings.
	Global disability	PT	2 RCTs (75 Ps)	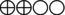 low	Downgraded due to low quality of SR despite a moderate level of evidence in the two RCTs.
	Emotional well‐being	PT	1 RCT (73 Ps)	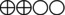 low	Downgraded because of indirectness due to different measurement instruments.
	Physical functioning	PT	3 RCTs (95 Ps), 1 CCT (78 Ps)	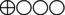 very low	Downgraded because of indirectness due to incomparable intervention settings, inconsistency of treatment effect and unclear RoB of one RCT.
Different group intervention approaches	Quality of life	SLT	3 RCTs (269 Ps)	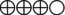 moderate	Downgraded to moderate because of partially inconsistent findings.
		PT	1 RCT (36 Ps)	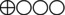 very low	Downgraded due to low number of Ps in one single study.
	Communication	SLT	5 RCTs (323 Ps), 1 CCT (37 Ps)	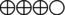 moderate	Downgraded to moderate because of partially inconsistent findings.
	Mobility	PT	1 SR (244 to 835 Ps), 1 CCT (16 Ps)	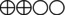 low	Low rating because of low quality of SR.
	Balance	PT	1 SR (103–190 Ps), 1 RCT (25 Ps), 1 CCT (16 Ps)	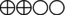 low	Low rating because of low quality of SR, and unclear RoB of RCT; indirectness because of incomparable interventions and inconsistency.
	Language	SLT	1 SR (142 Ps in meta‐analysis), 7 RCTs (360 Ps), 2 CCTs (57 Ps)	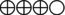 moderate	Downgraded to moderate because of high RoB of SR, unclear RoB of two RCTs and small inconsistencies in findings.
	Global disability	PT	1 SR (437 Ps), 1 RCT (42 Ps)	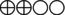 low	Downgraded because of low quality of SR.
	Emotional well‐being	SLT	2 RCT (53 Ps)	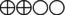 low	Downgraded to low because of partially inconsistent findings and evidence coming from only two studies with relatively small sample sizes.
	Physical functioning	PT	2 RCTs (62 Ps), 1 CCT (16 Ps)	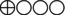 very low	Downgraded due to non‐randomized study design in two studies, inconsistency of resulting effects and indirectness because of heterogeneity of interventions.

Traumatic brain injury					
Comparison^b^	Outcome	Discipline	Number and types of studies (Ps)	Certainty of evidence (GRADE)	Comment
Group and individual intervention	Mobility	PT	1 RCT (65 Ps)	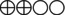 low	Downgraded to low because evidence comes from only one RCT.
	Balance	PT	1 RCT (65 Ps)	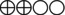 low	
	Global disability	PT	1 RCT (65 Ps)	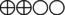 low	
	Emotional well‐being	PT	1 RCT (65 Ps)	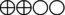 low	
	Communication	SLT	1 RCT (12 Ps)	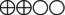 low	Downgraded to low because evidence comes from only one study with a small sample size.

.

Abbreviations: CCT, controlled clinical trial; GRADE, Grading of Recommendations Assessment, Development, and Evaluation; Ps, participants; PT, physical therapy; RCT, randomized controlled trial; RoB, risk of bias; SLT, speech‐language therapy; SR, systematic review; TBI, traumatic brain injury.

^a^ Certainty of evidence (GRADE): High = We are very confident that the true effect lies close to that of the estimate of the effect. Moderate = We are moderately confident in the effect estimate; the true effect is likely to be close to the estimate of the effect, but there is a possibility that it is substantially different. Low = Our confidence in the effect estimate is limited; the true effect may be substantially different from the estimate of the effect. Very low = We have very little confidence in the effect estimate; the true effect is likely to be substantially different from the estimate of effect.

^b^ For TBI, no data were available for the comparison groups “group and no intervention” and “different group intervention approaches.”

#### Intervention categories

Circuit class training was the most common PT intervention category and was found in four of the primary studies (44%),^12^, ^71^, ^72^, ^73^ and the included systematic review.^74^ The other eight studies used a synchronous group exercise or a mixed design.^65^ One trial added the group intervention to a multidisciplinary rehabilitation program.^65^ Two studies provided supplemental home exercises.^65^, ^75^ Most group interventions used land‐ or water‐based exercises; only one used imagery training.^75^ Two RCTs^62^, ^63^ focused on the effect of a constraint‐induced approach and one RCT^76^ used mirror therapy. The included review^74^ and four primary studies^73^, ^77^, ^78^, ^79^ focused on the lower limb, two on both extremities, and four on the upper limb, and four primary studies^62^, ^63^, ^65^, ^76^ targeted the upper limb.

All SLT studies addressed group treatment for neurogenic communication disorders. Except for one study on communicative‐pragmatic language impairment following TBI,^64^ all interventions targeted post‐stroke aphasia. The most frequently investigated group treatment approach was constraint‐induced aphasia therapy (CIAT),^80^, ^81^, ^82^, ^83^, ^84^, ^85^ an intensive form of SLT focusing on verbal production embedded in interactive and communicative language games. Other publications refer to this as intensive language‐action therapy (ILAT).^66^, ^80^, ^86^ As the studies on CIAT and ILAT included in this review all contained the same basic principles in their intervention descriptions, they are treated as representing the same approach (CIAT/ILAT) in the following. Some studies used CIATplus,^80^, ^81^, ^83^, ^85^, ^87^ an extended version, which adds written materials and involves relatives. The other SLT group intervention approaches were various forms of conversational group treatment,^67^, ^68^, ^88^ naming group treatment,^66^, ^89^ Mindfulness Meditation^70^ or multimodal group communication treatments.^7^, ^85^, ^87^, ^89^ One such multimodal approach, Multi‐Modality Aphasia Therapy (M‐MAT), is similar to CIAT and CIATplus but uses a different cueing strategy: When the target item cannot be produced verbally, the therapist provides additional cues, such as gestures, drawing, or reading the written word. The treatment was delivered face‐to‐face in all but two SLT studies with a non‐^67^ or semi‐immersive^68^ VR‐based conversational group treatment. Several studies supplemented the group treatment with additional home exercises^64^, ^70^, ^81^, ^83^, ^87^ or unlimited independent access to a VR platform outside of therapist‐supervised sessions.^67^

#### Outcomes and outcome measures

Trials from both disciplines used heterogeneous outcome measures from a variety of outcome domains that were mostly discipline specific (see Supplement B). Reported outcomes align with consensus statements on core outcome sets in neurorehabilitation.^90^, ^91^ SLT studies primarily measured communication and language, and PT studies included mobility, balance, physical function, and global disability. Only quality of life and emotional well‐being were measured in both disciplines.

#### Comparison groups

Across studies, group interventions were compared to no intervention, individual therapy, or alternative group approaches. In PT, three primary studies contrasted group‐based interventions with different group exercises^72^, ^75^, ^78^ and seven with individual treatment.^12^, ^62^, ^63^, ^71^, ^76^, ^77^, ^79^ The PT systematic review contained all three comparison groups.^74^ One three‐armed RCT compared two different group approaches and an individual intervention.^76^

In SLT, most studies compared group interventions with varying content^7^, ^64^, ^66^, ^68^, ^70^, ^83^, ^85^, ^89^ or intensity,^86^ three with individual therapy (usual care or intensive treatment),^81^, ^82^, ^88^ and two with no or a minimal amount of treatment.^67^, ^84^ Five studies used crossover designs to compare different group interventions.^7^, ^66^, ^67^, ^70^, ^85^ The SLT systematic review compared CIAT/ILAT to various controls^80^ but did not always distinguish between group and individual settings or outcome domains.

### Communication

Partially mixed evidence from one systematic review^80^ and four RCTs^67^, ^84^, ^86^, ^87^ with a total of 323 participants and an overall low certainty supports the assumption that SLT group interventions lead to gains in communication in aphasia when compared to no treatment. Although a systematic review^80^ reports better subjective communicative abilities after CIAT/ILAT compared to a nonintervention group, based on the results from one study, an RCT^84^ did not find the same effect. In another RCT,^86^ CIAT/ILAT resulted in improved communicative‐pragmatic performance. For CIATplus and M‐MAT, the results are inconsistent with benefits found for functional but not for multimodal communication in contrast to a group with no to minimal treatment.^87^ In a crossover RCT^67^ on non‐immersive VR‐based conversational group therapy, significant gains in functional communication were demonstrated, both in the group with immediate and deferred treatment. However, the same was not true for communication confidence.

When group treatment is compared to individual therapy, the evidence from two RCTs^83^, ^88^ and one CCT^81^ with a total of 134 participants is of low certainty and partially inconsistent. On the one hand, an RCT found a conversational group treatment to be more effective in improving gesture production than individual treatment.^88^ On the other hand, with regard to communicative effectiveness, individual usual care (UC) does not differ from CIAT/ILAT,^87^ and Model‐Orientated Aphasia Therapy (MOAT) does not differ from CIAT/ILAT or CIATplus.^81^ However, in the same CCT,^81^ higher communicative ratings were reported by patients and relatives for the MOAT than for the CIAT condition.

Studies comparing different SLT group treatment approaches indicate benefits with moderate‐certainty evidence based on five RCTs^7^, ^68^, ^86^, ^87^, ^89^ and one CCT^83^ with a total of 360 participants. One RCT^7^ found a multimodal group communication treatment superior to social group activities in enhancing functional communication – especially for severely affected patients. CIATplus appears to be at least equally effective or superior to CIAT/ILAT in improving communicative effectiveness according to one CCT.^83^ However, when CIATplus was contrasted with M‐MAT in an RCT,^87^ there was no difference in functional communication but multimodal communicative performance was significantly better after M‐MAT. An RCT^86^ comparing ILAT at different levels of intensity determined a difference between the highly and the moderately intense group with only the latter making further progress in the second of two training intervals. Group treatment with or without semi‐immersive VR in an RCT^68^ led to an equal increase in communicative ability, rated from the caregiver and the patient perspective. In contrast, neither a group with a specialized naming intervention nor a group with a non‐naming intervention (exercises in auditory comprehension, copying text, and reading) improved functional communication in an RCT.^89^

For patients with TBI, low‐certainty evidence from a single RCT^64^ with 12 participants addressing communicative‐pragmatic disorder suggests that a structured group communication program is effective in improving communicative skills when compared to a free conversation group.

### Language

When comparing group therapy with an untreated control condition, there is low‐certainty and partially inconsistent evidence from one systematic review^80^ and three RCTs^84^, ^86^, ^87^ with a total of 331 participants that the group treatment approaches have a beneficial effect on language. Whereas one study^84^ did not find a specific effect of CIAT on naming or any other language measure, another^86^ demonstrated that ILAT in a moderately or highly intense dose is beneficial to overall language performance in contrast to an untreated waiting period. CIATplus and M‐MAT, however, which additionally include written materials in the training, were shown to improve word retrieval of trained items in another RCT.^87^ One systematic review^80^ reports inconsistent results from a study comparing CIAT to a nonintervention group with no changes in any of the language measures.

In the comparison of SLT group interventions with intensity‐matched individual therapy, there is low‐certainty evidence from one systematic review,^80^ two RCTs,^82^, ^88^ and one CCT^81^ with a total of 154 participants that both lead to similar improvements. One RCT^88^ found UC to be superior to a conversational group treatment, with greater gains in overall linguistic performance and verbal production, whereas only participants in the conversational group treatment showed a comparatively better word fluency. In another RCT,^82^ patients receiving UC or CIAT showed an equal improvement in language, except for semantic fluency being better after UC and automatic speech being better after CIAT. A systematic review^80^ reports results from one study that found two patient groups to improve equally in language after UC or CIAT. One CCT^81^ contrasted MOAT in an individual setting with CIAT and CIATplus. The study found a significant difference only when MOAT was tested against CIAT, with MOAT leading to a greater improvement than CIAT in written language while MOAT and CIATplus were shown to be equally effective.

For the comparison of different SLT group approaches, there is evidence with an overall moderate‐certainty from one systematic review,^80^ seven RCTs,^7^, ^66^, ^68^, ^70^, ^86^, ^87^, ^89^ and two CCTs^67^, ^83^ with a total of 559 participants indicating an effect of high‐intense group interventions with CIAT/ILAT and CIATplus being equally effective or superior to other approaches such as stimulation aphasia therapy (SAT), M‐MAT, and intensive naming therapy (INT). In a crossover CCT,^70^ group‐based Mindfulness Meditation or mind wandering were equally ineffective in improving language. In contrast, individuals showed better overall linguistic performance in a crossover RCT^7^ after multimodal group communication treatment as opposed to social group activities. In another RCT,^89^ individuals with aphasia equally increased in naming trained items after a specialized naming group intervention and after a non‐naming group intervention with exercises in auditory comprehension, copying text, and reading. Comparing intensity‐matched group sessions of SAT, which involves multimodal exercises, with CIAT, a crossover RCT^85^ found a significant improvement in expressive language skills in both groups, especially in naming ability. The effect was higher for the group receiving CIAT first. When CIATplus was compared with M‐MAT in another RCT,^87^ CIATplus also resulted in greater gains in naming ability than M‐MAT. In a CCT,^83^ overall language performance improved equally in the CIAT and CIATplus condition. In another crossover RCT,^66^ ILAT led to a greater increase in language than INT. According to a further RCT,^86^ ILAT improved language performance independent of the extent of intensity. In a comparison of group conversational treatment with and without semi‐immersive VR scenarios, an RCT^68^ found that the linguistic skills of both groups improved equally. A systematic review^80^ reports language gains for different group treatments with no indication that CIAT is superior to approaches without constraint. This conclusion is supported by a meta‐analysis which pooled the performance of 109 patients and of 33 patients, respectively.

### Emotional well‐being

Regarding PT interventions after stroke, a low level of evidence based on one RCT^12^ with 73 participants indicates a non‐different effect for emotional well‐being comparing group and individual modalities. The RCT compared a group‐based exercise program with individual therapy and found no significant difference in the effect on symptoms of anxiety or depression.

For patients with TBI, there is low‐certainty evidence from a single RCT^65^ with 65 participants that combining UC with additional group treatment does not lead to a greater effectiveness: There was no difference in the effect on anxiety and depression between UC consisting of individual multidisciplinary outpatient rehabilitation and UC combined with group‐based vertigo rehabilitation and a home‐based exercise program.

In SLT, there is low‐certainty evidence from one RCT with 20 participants that perceived social isolation in poststroke aphasia can be reduced when a non‐immersive VR‐based conversational group treatment is compared to no treatment.^67^ In the comparison of different group approaches, evidence with low certainty from two RCTs with a total of 53 participants indicates an improvement in self‐esteem following conversational group treatment with or without a semi‐immersive VR^68^ and a positive effect of ILAT, but not of INT, on post‐stroke depression.^86^

### Mobility

Among the included PT studies on stroke survivor, outcomes related to mobility were particularly common, but the level of evidence was low in all comparison categories. One systematic review^74^ that pooled results from two studies with 269 participants indicates no advantage for circuit class training over no intervention. When contrasting group intervention with individual treatment, the evidence shows inconsistent effects for the outcome mobility based on four RCTs^12^, ^72^, ^77^, ^79^ with 145 participants and one CCT^71^ with 78 participants. Three RCTs found no difference in mobility outcome measures when comparing circuit class training with individual stretching and psychoeducation,^72^ group task‐oriented training with a home‐based walking exercise program,^77^ or group exercises containing self‐stretching, active range of motion exercises for upper and lower limb, and minimal gait training with individual gait training using a robotic device.^79^ In contrast, one RCT^12^ showed circuit class therapy to be more effective in improving walking ability than individual exercises. The results of one CCT,^71^ comparing circuit class training with individual UC, were mixed. Both groups showed similar improvements in upper‐limb function at all measured time points, and walking capacity improved equally in both groups by the end of the intervention. After completing circuit class training, participants required less assistance than those in individual care. However, at 6‐month follow‐up, patients who received individual treatment demonstrated better walking capacity.

Studies comparing different group intervention approaches yielded low‐certainty evidence with inconsistent results based on one systematic review^74^ with 244 to 835 participants and one CCT^75^ with 16 participants. The systematic review^74^ found circuit class training to be superior to other group interventions after pooling results for several outcome measures of functional mobility (walking capacity, gait speed, risk of falls) and physical activity. In contrast, one CCT^75^ found no significant difference between two variants of group‐based motor imagery training with kinesthetic or visual focus.

For patients with TBI, there is low‐certainty evidence from a single RCT^65^ with 65 participants that group treatment in addition to UC is more effective than UC alone: Mobility improved significantly more when UC consisting of individual multidisciplinary outpatient rehabilitation was combined with group‐based vertigo rehabilitation and a home‐based exercise program.

### Balance

Very‐low‐certainty evidence based on three RCTs^12^, ^77^, ^79^ with 175 participants shows inconsistent effects for PT group therapy in patients with stroke compared with individual therapy. One RCT^79^ found an individual intervention involving a robotic leg brace to be superior to group sessions with self‐stretching, active range of motion exercises for upper and lower limb, and minimal gait training after 6 weeks but not at the 3 months follow‐up. The other RCT^12^ and a CCT^71^ could not determine a difference between circuit class therapy and individual treatment or individual UC at either posttest or follow‐up.

When comparing different PT group intervention approaches for stroke survivors, a low level of evidence based on one systematic review^74^ with 103–190 participants, an RCT^78^ with 25 participants, and a CCT^75^ with 16 participants showed inconsistent effects. The meta‐analysis of the included systematic review,^74^ in which the results of four studies with 171 participants, three with 190 participants, and another four with 103 participants were pooled for three different outcome measures, could not determine a clear superiority of circuit class training over other group approaches: Although no difference was found for two of the measures, the third indicated a significant effect in favor of the circuit class intervention. An RCT^78^ found a significantly greater improvement after an aquatic group exercise than after a land‐based group exercise program. In a CCT,^75^ no significant differences were shown between a motor imagery group with kinesthetic focus or with visual focus.

For patients with TBI, there is low‐certainty evidence from a single RCT^65^ with 65 participants that combining UC with additional group treatment does not lead to a greater effectiveness: There was no difference in the effect on balance between UC consisting of individual multidisciplinary outpatient rehabilitation and UC combined with group‐based vertigo rehabilitation and a home‐based exercise program.

### Physical function

A very low level of evidence based on three RCTs^62^, ^76^, ^79^ with 95 participants and one CCT^71^ with 78 participants shows inconsistent effects for the comparison of PT group versus individual intervention. One RCT^62^ found constraint‐induced movement therapy (CIMT) in a group setting to be more effective in improving upper‐limb function than in an individual setting. However, in another RCT,^76^ finger motor function showed significantly greater improvement following individual mirror therapy compared to group mirror therapy; no difference was found for other measures of UE function. No significant effect for lower‐limb function was found in an RCT^79^ for individual gait training with a robotic brace compared to group sessions with self‐stretching, active range of motion exercises, and minimal gait training.

In the comparison group with different PT group interventions, evidence with very low certainty from two RCTs^76^, ^78^ with 62 participants and one CCT^75^ with 16 participants could not show significant differences between the approaches. An RCT^76^ contrasting group mirror therapy with sham mirror therapy did not find a significant difference for UE function. In another RCT,^78^ no difference was determined in everyday motor function when a water‐ and a conventional land‐based group therapy was compared. However, the change in the torque of the knee flexor on the affected side was greater after aquatic group therapy than after conventional group therapy. For unilateral gross manual dexterity, no difference was determined between group‐based motor imagery with kinesthetic or visual focus in a CCT.^75^

### Global disability

When PT group interventions are compared to individual therapy in patients with stroke, there is evidence with low certainty based on two RCTs^63^, ^76^ with 75 participants. One study^76^ found no significant difference in the effect on global disability between group and individual mirror therapy. The other^63^ favored CIMT in a group setting over individual CIMT for improving global disability. However, cognitive outcomes improved significantly more after group training, whereas individual therapy resulted in greater gains in the motor dimension.

Low‐certainty evidence based on one systematic review^74^ with 437 participants and one RCT^76^ with 42 participants indicates inconsistent effects. Although the meta‐analysis of the systematic review^74^ that pooled results from studies with 437 participants showed a superior effect of circuit class therapy over other kinds of PT group therapy, an RCT found no difference in the effect on global disability between group mirror therapy and sham mirror therapy in the same setting.

For TBI, a single RCT^65^ provides low‐quality evidence that combining individual UC with a group intervention improves global disability, though benefits were limited to short‐term follow‐up.^34^

## DISCUSSION

This systematic review summarized and critically appraised group treatment in neurorehabilitation in PT and SLT. Further, it aimed at gathering evidence on interdisciplinary approaches and the transfer of group‐based treatments to a telerehabilitation setting. A total of 29 publications were identified as meeting the eligibility criteria, 16 thereof addressing group treatment in SLT (1 systematic review, 12 RCTs, and 3 CCTs with a total of 861 participants) and 13 in PT (1 systematic review, 9 RCTs and 3 CCTs with a total of 1757 participants). We found no studies that implemented an interdisciplinary treatment integrating two disciplines. Only two studies investigated interventions suitable for telerehabilitation, both in the field of SLT and involving VR.

There is some evidence suggesting that monodisciplinary group treatments from SLT and PT are an effective form of neurorehabilitation, but the available data vary significantly and are insufficient to draw a final conclusion. When compared to no therapy, PT group therapy shows no greater effect on mobility in patients with stroke.^73^, ^74^ The comparison of group therapy with individual treatment in stroke survivors showed a non‐different effect for the outcomes quality of life^77^ and emotional well‐being.^12^, ^76^ For the outcomes mobility,^12^, ^71^, ^72^, ^77^, ^79^ balance,^12^, ^77^, ^79^ physical function,^62^, ^71^, ^76^, ^79^ and global disability^63^, ^76^ inconsistent results were found. When different group treatments are compared, effects vary according to the specific approach: Circuit class training compared to other forms of group therapy is superior in improving quality of life,^72^ mobility,^71^ and global disability,^71^ whereas a non‐different effect was shown for balance.^71^ Water‐based group therapy demonstrates superior balance outcomes compared to land‐based therapy, though physical function results are similar.^78^ No differences were observed in mobility, balance, or physical function between kinesthetic and visual focus in motor imagery.^75^ Similarly, group mirror therapy shows no advantage over group sham mirror therapy for physical function, emotional well‐being, or global disability.^76^ Patients with TBI experience short‐ but no long‐term benefits in mobility and global disability from combining individual UC with an additional group intervention.^65^

SLT group treatment appears to be an effective therapy for individuals with post‐stroke aphasia and cognitive‐pragmatic language impairment following TBI, with the evidence base for the first group of patients being a lot broader than for the second. Benefits were found for different outcomes and approaches, which mostly followed an intense training schedule. However, a definite conclusion cannot be drawn because the evidence is somewhat inconsistent, the certainty of evidence varies, and not all approaches have been investigated in all comparison groups. Although there is mostly low‐certainty evidence that group therapy is beneficial when compared to no^67^, ^70^, ^80^, ^84^, ^86^, ^87^ or individual treatment,^81^, ^82^, ^88^ moderate‐certainty evidence indicates that some group treatment approaches are equally effective or superior to others in people with aphasia with regards to quality of life, communication, and language: M‐MAT appears to be more effective than CIATplus in improving quality of life and communicative skills,^87^ which in turn might be superior to CIAT/ILAT.^83^ However, CIAT/ILAT appears to have greater benefits for language than INT and SAT^66^, ^85^ and for emotional well‐being than INT.^66^ As to communication or language gains, there seems to be no difference between a conversational group with and without semi‐immersive VR.^68^ Taken together, group treatments appear to be most beneficial when targeting verbal production in communicative settings, especially when supplemented with written materials. However, using a multimodal cueing strategy that incorporates nonverbal elements such as gestures and drawing to facilitate verbal expression may be more effective than a strict constraint on the verbal modality.

To address heterogeneity in the outcomes of the reviewed studies, we used core outcome sets^90^, ^91^ to structure our results and synthesize findings in a clinically meaningful and comparable way. While this heterogeneity reduces generalizability, it also provides valuable insights: it underscores the need for greater methodological standardization and highlights the considerable evidence gap for interdisciplinary group interventions. Despite the longstanding call for interdisciplinary research,^92^ no integrated group approach was found. This is surprising, particularly for a collaborative approach of SLT and PT, given the benefits of aerobic exercise to neuroplastic change^40^, ^41^ and the neurofunctional overlap of motor and speech/language areas.^30^ There is still no “combinatorial hand‐arm‐language paradigm” in rehabilitation as demanded by Wortman‐Jutt and Edwards^93^ despite the evidence indicating cross‐domain functional improvement and synergistic effects.^34^, ^35^, ^38^, ^39^, ^42^ An integrated SLT/PT group approach could draw from shared principles, as the most common approach in SLT groups found in this review were forms of CIAT, which is rooted in CIMT in PT.^94^ Only two pilot trials, not meeting our eligibility criteria, found preliminary evidence indicating the feasibility and potential beneficial effect of an online group neurorehabilitation involving two or more disciplines: Whereas one approach can be considered interdisciplinary – integrating SLT and PT into a single treatment targeting both motor and speech/language impairments after stroke^46^, ^47^ – the other represents a multidisciplinary model, in which separate online groups are conducted by therapists from different professions.^16^, ^95^

Aiming at expanding interdisciplinary collaboration of PT and SLT, certain syndromes with neuromotor impairments, such as dysarthria, dysphagia, and apraxia of speech, create an interesting interface to build joint treatment programs. Although there already are group‐based approaches to address dysarthria – mostly in neurodegenerative diseases such as Parkinson's (e.g., Edwards et al.^96^), which were not the subject of this review – we did not find any study on group therapy in apraxia of speech and only one review, not meeting our eligibility criteria, mentioned group training as a setting for dysphagia treatment.^97^

With regard to other interdisciplinary approaches, our findings indicate the potential for a further development of the collaboration between speech and language pathologists and psychologists in the treatment of neurogenic communication disorders and associated sequelae such as post‐stroke depression.^98^, ^99^ We found some group approaches combining art^100^ and drama therapy^101^ with SLT or music therapy with either SLT^102^ or PT.^103^ The fact that these studies did not meet our methodological eligibility criteria once again demonstrates the need for more high‐quality research. Although interdisciplinary treatment approaches are recommended in neurorehabilitation guidelines,^1^, ^3^ interprofessional collaboration in clinical practice is still considered insufficient^104^, ^105^ and attributed to a reductionists view and the overspecialization in the medical field.^31^, ^106^ Thus, interdisciplinary communication and collaboration in research and practice needs to be promoted with the aim of “putting the patient back together”^107^ and thereby applying a holistic approach^34^ toward the patients' needs as framed by the biopsychosocial model of the ICF.^24^, ^25^, ^27^ However, in order to facilitate the clinical implementation of interdisciplinary group approaches, adjustments to the billing systems may be needed, as not all countries reimburse multiple therapists involved in one therapy session.^108^, ^109^, ^110^, ^111^

There is also a need for further research on group treatment approaches delivered in a telerehabilitation setting as we merely found two studies on group treatments suitable for an implementation in telerehabilitation, both using non‐^67^ or semi‐immersive^67^, ^68^ VR in SLT group interventions. Although several systematic reviews conclude that there is preliminary evidence for the effectiveness of telerehabilitation in neurologic patients,^19^, ^112^, ^113^, ^114^, ^115^ these include only individual treatment studies. This leaves insufficient data from pilot studies on other forms of telerehabilitation such as group video therapy.^16^, ^18^

This lack of high‐quality evidence must be addressed to ensure sustainable healthcare in the decades to come, particularly as effective telerehabilitation becomes an integral part of service delivery. Implementing telerehabilitation offers opportunities for cost savings by reducing travel distances, increasing training frequency, and facilitating the involvement of relatives in the treatment process.^116^

## LIMITATIONS

Electronic searches were challenging due to varied terminology for group treatments (eg, “group therapy,” “group intervention,” “group‐based approach,” “group exercise” etc.) and frequent use of the word “group” in study design descriptions (eg, “intervention group,” “control group”). We may have missed some relevant studies published in languages other than English or German due to our language restrictions. By summarizing the results according to established core outcome sets, some details on subdomains of the reported outcomes (eg, the different language modalities included in the outcome “language”) may have been lost in favor of a more general statement.

## CONCLUSION

Group treatments in neurorehabilitation show some benefits, but further research is needed, especially regarding interdisciplinary approaches and telerehabilitation.

## FUNDING INFORMATION

The review was partially conducted during the project “YourHouse 4.0 Upper Palatinate” funded by the Bavarian State Ministry of Health, Care and Prevention.

## DISCLOSURE

The authors declare no potential conflicts of interest with respect to the research, authorship, and/or publication of this article.

## PREREGISTRATION

The review was preregistered with International Prospective Register of Systematic Reviews (CRD42021288012).

This journal‐based CME activity is designated for 1.0 *AMA PRA Category 1 Credit* ^TM^. Effective January 2024, learners are no longer required to correctly answer a multiple‐choice question to receive CME credit. Completion of an evaluation is required, which can be accessed using this link, https://onlinelearning.aapmr.org/. This activity is FREE to AAPM&R members and available to nonmembers for a nominal fee. CME is available for 3 years after publication date. For assistance with claiming CME for this activity, please contact (847) 737–6000. All financial disclosures and CME information related to this article can be found on the Online Learning Portal (https://onlinelearning.aapmr.org/) prior to accessing the activity.

## Supporting information


Supplementary A

Supplementary B

Supplementary C

Supplementary D

## Data Availability

All data used in the systematic review will be shared upon request. The central data can be found in the submitted supplementary files (Supplement B, C, and D).
