# Supplementary material for: Group rehabilitation for adults with acquired neurological disorders: A systematic review of mono‐ and interdisciplinary interventions in physical and speech‐language therapy
Source: PM R. 2025 Nov 11;18(3):315–31. doi: 10.1002/pmrj.70006 (PMC13001142; doi:10.1002/pmrj.70006)
Supplement: Supplementary file 1 — Supplementary A [file PMRJ-18-315-s003.pdf]

Supplement A: List of acronyms

Acronym	Full version
AMSTAR	A Measurement Tool to Assess Systematic Reviews
CCT	Controlled Clinical Trial
CIAT	Constraint-Induced Aphasia Therapy
CIMT	Constraint-Induced Movement Therapy
GRADE	Grading of Recommendations Assessment, Development and Evaluation
ICF	International Classification of Functioning, Disability and Health
ILAT	Intensive Language-Action Therapy
INT	Intensive Naming Therapy
JBI	Joanna Briggs Institute
LSVT	Lee Silverman Voice Treatment
M-MAT	Multi-Modality Aphasia Therapy
MOAT	Model-Orientated Aphasia Therapy
N-ROL	Neuro-Rehabilitation OnLine
PEDro	Physiotherapy Evidence Database
PRISMA	Preferred Reporting Items for Systematic Reviews and Meta-Analyses
PT	Physical Therapy
RCT	Randomized Controlled Trial
SAT	Stimulation Aphasia Therapy
SLT	Speech-Language Therapy
TBI	Traumatic Brain Injury
UC	Usual Care
UE	Upper Extremity
VR	Virtual Reality
WFNR	World Federation of Neurorehabilitation
