# Supplementary material for: Group rehabilitation for adults with acquired neurological disorders: A systematic review of mono‐ and interdisciplinary interventions in physical and speech‐language therapy
Source: PM R. 2025 Nov 11;18(3):315–31. doi: 10.1002/pmrj.70006 (PMC13001142; doi:10.1002/pmrj.70006)
Supplement: Supplementary file 2 — Supplementary B [file PMRJ-18-315-s002.pdf]

Supplement B: Search strings used in the databases PubMed, CINAHL and Cochrane

PubMed

("central nervous system diseases"[MeSH Terms] OR "cranial nerve diseases"[MeSH Terms] OR "neurologic manifestations"[MeSH Terms] OR "trauma, nervous system"[MeSH Terms] OR "stroke"[Text Word] OR "cerebrovascular"[Text Word] OR "apople*"[Text Word] OR (("brain"[Text Word] OR "cerebral"[Text Word] OR "intracerebral"[Text Word] OR "intracranial"[Text Word] OR "cranial nerve"[Text Word] OR "spinal cord"[Text Word]) AND ("infarction"[Text Word] OR "ischemia"[Text Word] OR "hemorrhage"[Text Word] OR "trauma*"[Text Word] OR "injur*"[Text Word] OR "lesion"[Text Word] OR "diseas*"[Text Word] OR "palsy"[Text Word] OR "deficit*"[Text Word])) OR (("vertebral"[Text Word] OR "cervical"[Text Word]) AND "artery dissection"[Text Word]))

AND

("spasm*"[Text Word] OR "spastic"[Text Word] OR "atactic"[Text Word] OR "ataxia*"[Text Word] OR "paresis"[Text Word] OR "paretic"[Text Word] OR "hemiplegi*"[Text Word] OR "hemipare*"[Text Word] OR "sensorimotor*"[Text Word] OR "aphasi*"[Text Word] OR "dysarthri*"[Text Word] OR (("speech"[Text Word] OR "verbal"[Text Word]) AND "apraxi*"[Text Word]))

AND

("physical therapy modalities"[MeSH Terms] OR "exercise"[MeSH Terms] OR "exercise therapy"[MeSH Terms] OR "physical examination"[MeSH Terms] OR "postural balance"[MeSH Terms] OR "gait"[MeSH Terms] OR "exercis*"[Text Word] OR "physical therap*"[Text Word] OR "physiotherap*"[Text Word] OR "rehabilitation of speech and language disorders"[MeSH Terms] OR (("dexterity"[Text Word] OR "postural"[Text Word] OR "gait*"[Text Word] OR "balance*"[Text Word] OR "motricit*"[Text Word] OR "walk*"[Text Word] OR "aerobic capacit*"[Text Word] OR "mobili*"[Text Word] OR "movement"[Text Word] OR "muscle strength*"[Text Word] OR "functional independen*"[Text Word] OR "function independen*"[Text Word] OR "daily living"[Text Word] OR "daily life"[Text Word] OR "speech"[Text Word] OR "language"[Text Word] OR "communicat*"[Text Word] OR "conversation*"[Text Word] OR "linguistic*"[Text Word]) AND ("therap*"[Text Word] OR "intervention*"[Text Word] OR "train*"[Text Word] OR "rehabilit*"[Text Word])))

AND

("self management"[MeSH Terms] OR "self care"[MeSH Terms] OR "self help groups"[MeSH Terms] OR "social support"[MeSH Terms] OR "group*"[Text Word])

AND

(("clinical"[Title/Abstract] AND "trial"[Title/Abstract]) OR ("clinical trials as topic"[MeSH Terms] OR "clinical trial"[Publication Type] OR "random*"[Title/Abstract] OR "random allocation"[MeSH Terms] OR "therapeutic use"[MeSH Subheading]) OR ("systematic review"[Title] OR "systematic literature review"[Title] OR "systematic scoping review"[Title] OR "systematic narrative review"[Title] OR "systematic qualitative review"[Title] OR "systematic evidence review"[Title] OR "systematic quantitative review"[Title] OR "systematic meta review"[Title] OR "systematic critical review"[Title] OR "systematic mixed studies review"[Title] OR "systematic mapping review"[Title] OR "systematic cochrane review"[Title] OR "systematic search and review"[Title] OR "systematic integrative review"[Title]))

Filter

PubMed Therapy Filter: sensitive/broad:

((clinical[Title/Abstract] AND trial[Title/Abstract]) OR clinical trials as topic[MeSH Terms] OR clinical trial[Publication Type] OR random*[Title/Abstract] OR random allocation[MeSH Terms] OR therapeutic use[MeSH Subheading])

PubMed Systematic Reviews Filter (adapted version):

(systematic review[ti] OR systematic literature review[ti] OR systematic scoping review[ti] OR systematic narrative review[ti] OR systematic qualitative review[ti] OR systematic evidence review[ti] OR systematic quantitative review[ti] OR systematic meta-review[ti] OR systematic critical review[ti] OR systematic mixed studies review[ti] OR systematic mapping review[ti] OR systematic cochrane review[ti] OR systematic search and review[ti] OR systematic integrative review[ti])

CINAHL

(MH "central nervous system diseases+" OR MH "cranial nerve diseases+" OR MH "neurologic manifestations+" OR TX "stroke" OR TX "cerebrovascular" OR TX "apople*" OR ((TX "brain" OR TX "cerebral" OR TX "intracerebral" OR TX "intracranial" OR TX "cranial nerve" OR TX "spinal cord") AND (TX "infarction" OR TX "ischemia" OR TX "hemorrhage" OR TX "trauma*" OR TX "injur*"OR TX "lesion" OR TX "diseas*" OR TX "palsy" OR TX "deficit*")) OR ((TX "vertebral" OR TX "cervical") AND TX "artery dissection"))

AND

(TX "spasm*" OR TX "spastic" OR TX "atactic" OR TX "ataxia* OR TX "paresis" OR TX "paretic" OR TX "hemiplegi*" OR TX "hemipare*" OR TX "sensorimotor*" OR TX "aphasi*" OR TX "dysarthri*" OR ((TX "speech" OR TX "verbal") AND TX "apraxi*"))

AND

(MH "physical therapy+" OR MH "exercise" OR MH "physical examination+" OR MH " balance, postural+ " OR MH "gait+" OR TX "exercis*" OR TX "physical therap*" OR TX "physiotherap*" OR MH "rehabilitation, speech and language+" OR ((TX "dexterity" OR TX "postural" OR TX "gait*" OR TX "balance*" OR TX "motricit*" OR TX "walk*" OR TX "aerobic capacit*" OR TX "mobili*" OR TX "movement" OR TX "muscle strength*" OR TX "functional independen*" OR TX "function independen*" OR TX "daily living" OR TX "daily life" OR TX "speech" OR TX "language" OR TX "communicat*" OR TX "conversation*" OR TX "linguistic*") AND (TX "therap*" OR TX "intervention*" OR TX "train*" OR TX "rehabilit*"))))

AND

(MH "self-management+" OR MH "self care+" OR MH "support groups+" OR MH "support, psychosocial+" OR TX "group*")

AND

((TI ("clinical" AND "trial") OR AB ("clinical" AND "trial") OR MH "clinical trials+" OR PT "clinical trial" OR TI "random*" OR AB "random*" OR MH "random assignment" OR (TI "systematic review" OR TI "systematic literature review" OR TI "systematic scoping review" OR TI "systematic narrative review"

OR TI "systematic qualitative review" OR TI "systematic evidence review" OR TI "systematic quantitative review" OR TI "systematic meta review" OR TI "systematic critical review" OR TI "systematic mixed studies review" OR TI "systematic mapping review" OR TI "systematic cochrane review" OR TI "systematic search and review" OR TI "systematic integrative review"))

Cochrane

([mh "central nervous system diseases"] OR [mh "cranial nerve diseases"] OR [mh "neurologic manifestations"] OR [mh "trauma, nervous system"] OR "stroke" OR "cerebrovascular" OR "apople*" OR (("brain" OR "cerebral" OR "intracerebral" OR "intracranial" OR "cranial nerve" OR "spinal cord") AND ("infarction" OR "ischemia" OR "hemorrhage" OR "trauma*" OR "injur*" OR "lesion" OR "diseas*" OR "palsy" OR "deficit*")) OR (("vertebral" OR "cervical") AND "artery dissection"))

AND

("spasm*" OR "spastic" OR "atactic" OR "ataxia*" OR "paresis" OR "paretic" OR "hemiplegi*" OR "hemipare*" OR "sensorimotor*" OR "aphasi*" OR "dysarthri*" OR (("speech" OR "verbal") AND "apraxi*"))

AND

([mh "physical therapy modalities"] OR [mh "exercise"] OR [mh "exercise therapy"] OR [mh "physical examination"] OR [mh "postural balance"] OR [mh "gait"] OR "exercis*" OR "physical therap*" OR "physiotherap*" OR [mh "rehabilitation of speech and language disorders"] OR "dexterity" OR "postural" OR "gait*" OR "balance*" OR "motricit*" OR "walk*" OR "aerobic capacit*" OR "mobili*" OR "movement" OR "muscle strength*" OR "functional independen*" OR "function independen*" OR "daily living" OR "daily life" OR "speech" OR "language" OR "communicat*" OR "conversation*" OR "linguistic*") AND ("therap*" OR "intervention*" OR "train*" OR "rehabilit*")))

AND

([mh "self management"] OR [mh "self care"] OR [mh "self help groups"] OR [mh "social support"] OR "group*")

AND

(("clinical":ti AND "trial":ti) OR ("clinical":ab AND "trial":ab) OR ([mh "clinical trials as topic"] OR "clinical trial":pt OR "random*":ti OR "random*":ab OR [mh "random allocation"] OR [mh "therapeutic use"]) OR ("systematic review":ti OR "systematic literature review":ti OR "systematic scoping review":ti OR "systematic narrative review":ti OR "systematic qualitative review":ti OR "systematic evidence review":ti OR "systematic quantitative review":ti OR "systematic meta review":ti OR "systematic critical review":ti OR "systematic mixed studies review":ti OR "systematic mapping review":ti OR "systematic cochrane review":ti OR "systematic search and review":ti OR "systematic integrative review":ti))
