# Supplementary material for: Group rehabilitation for adults with acquired neurological disorders: A systematic review of mono‐ and interdisciplinary interventions in physical and speech‐language therapy
Source: PM R. 2025 Nov 11;18(3):315–31. doi: 10.1002/pmrj.70006 (PMC13001142; doi:10.1002/pmrj.70006)
Supplement: Supplementary file 3 — Supplementary C [file PMRJ-18-315-s001.pdf]

Supplement C: Certainty of evidence (GRADE)

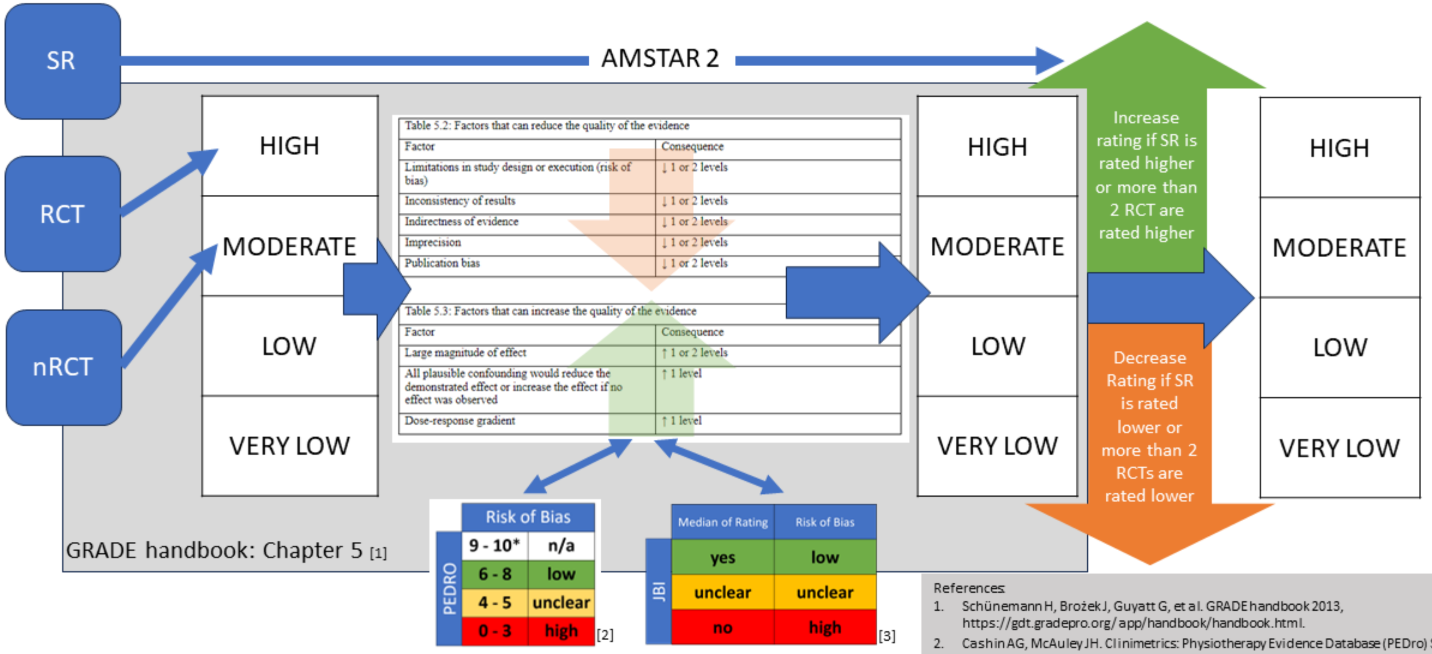

* A PEDro score of 9-10 is considered excellent quality with a very low risk of bias[2], but is not applicable for most intervention studies as blinding of subjects and therapists is not feasible.

References:
1. Schünemann H, Brożek J, Guyatt G, et al. GRADE handbook 2013, https://gdt.gradepro.org/ app/handbook/handbook.html.
2. Cashin AG, McAuley JH. Clinimetrics: Physiotherapy Evidence Database (PEDro) Scale. *J Physiother* 2020; 66: 59.
3. Barker TH, Habibi N, Aromataris E, Stone JC, Leonardi-Bee J, Sears K, et al. The revised JBI critical appraisal tool for the assessment of risk of bias quasi-experimental studies. JBI Evid Synth. 2024;22(3):378-88.

Figure 1: Concept for the determination of certainty of evidence
