# Supplementary material for: Group rehabilitation for adults with acquired neurological disorders: A systematic review of mono‐ and interdisciplinary interventions in physical and speech‐language therapy
Source: PM R. 2025 Nov 11;18(3):315–31. doi: 10.1002/pmrj.70006 (PMC13001142; doi:10.1002/pmrj.70006)
Supplement: Supplementary file 4 — Supplementary D [file PMRJ-18-315-s004.pdf]

Supplement D: Study characteristics

Table 1: Overview of PT and SLT studies

**Discipline**	**Study designs**	**Study locations**	**Publication years**	**Number of participants**	**Treatment dose**	**Setting of intervention**	**Mode of delivery**	**Participants & therapist/group session**	**Professions involved**
PT	Primary studies: n = 12<br>- RCTs: n = 9<br>- CCTs: n = 3	Primary studies:<br>- Australia: n = 1<br>- Brazil: n = 2<br>- Chile: n = 2<br>- Germany: n = 2<br>- Italy: n = 1<br>- Korea: n = 1<br>- Norway: n = 1<br>- Sweden: n = 1<br>- USA: n = 1	2006-2020	Primary studies:<br>- total: n = 437 (6 - 40; M = 228.7; SD = 11.1)<br>- IG: n = 228 (6 - 38; M = 21.8; SD = 10.3)<br>- CG: n = 209 (6- 40; M = 21.0; SD = 11,9)	Primary studies:<br>- IG: 308.0 total hours (5.0 – 60.0; M = 30.33; SD = 15.0)<br>- CG: 244.0 total hours (0.0 – 54.0; M = 23.2; SD = 17,1)	- inpatient: n = 3<br>- outpatient: n = 6<br>- no information/ unclear: n = 2	Primary studies:<br>- face-to-face: n = 12<br>- non-immersive VR: n = 0<br>semi-immersive VR: n = 0	Primary studies:<br>- participants: >2<br>- therapist(s): n = unclear	Primary studies:<br>PT: n = 8<br>PT plus assistant/ student: n = 4
	Systematic review: n = 1	Systematic review:<br>- Australia: n = 1		Systematic review:<br>- Total: n = 1320 (4-126); M = 753.61; SD = 37,7.<br>- IG: n = 656 (5 – 126; M = 38.6; SD = 38.0)<br>- CG: n = 664.0 (4 – 124; M = 39.1; SD = 38.2)	Systematic review:<br>Inconsistently reported in Holmgren 2010 - Only calculated with published data:<br>- IG: 476.6 total hours (6.0 – 72.0; M = 29.8; SD = 18.9)<br>CG: 403.4 total hours (0 – 72.0; M = 23.73; SD = 21.0)	Systematic review:<br>- inpatient: n* = 6<br>- outpatient: n* = 11<br>no information/ unclear: n* = 0	Systematic review:<br>- face-to-face: n* = 17<br>- non-immersive VR: n* = 0<br>semi-immersive VR: n* = 0	Systematic review:<br>more than two participants per therapist	Systematic review:<br>PT: n* = 6<br>PT plus assistant/ student/ volunteer: n* = 4<br>Multidisciplinary: * = 3<br>Therapist/ Instructor: n* = 2<br>PT + OT: n* = 1<br>PT + sport therapist: n* = 1
SLT	Primary studies: n = 15<br>- RCTs: n = 12<br>- CCTs: n = 3	- Australia/New Zealand: n = 1<br>- China: n = 1<br>- Germany: n = 3<br>- Germany/Switzerland: n = 1<br>- Italy: n = 1	1981 - 2022	Primary studies:<br>- total: n = 589 (8 - 216; M = 41.2; SD = 49.2)<br>- IG: n = 312 (5 - 146; M = 21.5; SD = 34.0)<br>- CG: n = 286 (3 - 70; M = 19.8; SD = 17.2)	Primary studies:<br>- IG: 1.6 - 176 h (M = 47.7; SD = 40.5)<br>- CG: 0 - 176 h (M = 31.0; SD = 38.3)	- inpatient: n = 5<br>- outpatient: n = 6<br>- home: n = 1<br>- no information: n = 4	- face-to-face: n = 14<br>- non-immersive VR: n = 1<br>- semi-immersive VR: n = 1	- participants: n = 2-7<br>- therapist(s): n = 1-2	- SLP(s): n = 13<br>- SLP plus student/volunteer: n = 2<br>- SLP plus performing artist: n = 1

	Systematic review: n = 1	- Portugal: n = 1<br>- Serbia: n = 1<br>- Sweden: n = 1<br>- UK: n = 1<br>- USA: n = 4	Systematic review:<br>- total: n = 272 (18-153; M = 90.7; SD = 55.6)<br>- IG: n = 152 (9 – 79; M = 50.7; SD = 30.1)<br>- CG: n = 120 (9 – 74; M = 40; SD = 26.6)	Systematic review:<br>- IG: 15 - 30 h (M = 27; SD = 7.4)<br>- CG: 0 – 30 h (M = 21.4; SD = 8.0)

*number of included primary studies in the review

Table 2: Summary of SLT primary studies

ID	Study	Design	Etiology	Syndrome/ severity	Sample size	Intervention	Length/frequency	Outcome measures	Main result
64	Agrela et al. (2021)	RCT	TBI	Moderate to severe TBI with communicative-pragmatic language impairment	IG: n = 6	IG: Structured group communication program based on the Cognitive pragmatic treatment and the Group interactive structured treatment (GIST), 6 participants & 2 SLPs/group session	24 sessions of 1.5 h each, twice a week, for 12 weeks, 36 h in total	Assessment Battery of Communication (ABaCo), functional assessment of daily communication by family member/caregiver	Greater improvement regarding comprehension and production of facial expressions, speech intonation and gesture production with structured group communication; gains in the production of facial expressions and speech intonation maintained at 3- months follow-up; significantly better rated daily communication at follow-up compared to baseline
					CG: n = 6	CG: Free conversation group, 6 participants & 2 SLPs/group session			
81	Barthel et al. (2008)	CCT	Stroke	Mild to severe aphasia	IG: n = 12	IG: Intensive model-orientated aphasia therapy (MOAT) in individual setting	10 sessions on 10 consecutive days, 3 hours/day, 30 h in total	Aachen Aphasia Test (AAT), Communicative Effectiveness Index (CETI), Communicative Activity Log (CAL), picture naming task, Lexikon modellorientiert (Lexicon model-oriented – LEMO), Wortproduktionsprüfung (Word Production Test),	Greater improvement in language function and communicative ability with MOAT compared to CIAT; equally great gains when MOAT is compared to CIATplus; generalization of naming performance to untrained items in MOAT group; stable effects over 6 months follow-up period
					CG: n = 27 (CIAT n = 12; CIATplus n = 15)	CG: Constraint-induced aphasia therapy (CIAT) or CIATplus, 2-3 participants & 2 SLPs/group session			

ID	Study	Design	Etiology	Syndrome/ severity	Sample size	Intervention	Length/fre- quency	Outcome measures	Main result
								Pyramids and Palm Trees Test	
7	Elman & Bernstein-Ellis (1999)	Crosso-ver RCT	Stroke	Mild to se-vere apha-sia	IG: n = 12	IG: Immediate multimodal group communication treat-ment; 7 participants & 1 SLP plus 1 student/volun-teer/group session; ½ ses-sion/week co-led by per-forming artist	IG: 32 sessions of 2,5 h each, twice a week, for 4 months, 80 h in total	Shortened Porch Index of Communicative Abilities (SPICA), Western Aphasia Battery – Aphasia Quo-tient (WAB AQ), Commu-nicative Abilities in Daily Living (CADL)	Higher scores on communicative and linguistic measures for participants receiving group communication treatment after 2 and 4 months of treatment; effects maintained at 4-6-weeks follow-up
					CG: n = 12	CG: Deferred treatment; engagement in social group activities while awaiting treatment	CG: Before in-tervention ≥ 3 h/week of wait-list program, for 4 months, ≥ 48 h in total		
68	Giachero et al. (2020)	RCT	Stroke	Moderate nonfluent aphasia	IG: n = 18	IG: Conversational group treatment using semi-im-mersive VR scenarios, 3 participants & 1 SLP/group session	48 sessions of 1 h each, twice a week for 24 weeks, 48 in to-tal[1]	Aachen Aphasia Test (AAT), Conversation Anal-ysis Profile for People with Aphasia test (CAPPA test), Visual Analogue Self-Esteem Scale (VASES), World Health Or-ganization Quality of Life Scale (WHOQoL)	Both groups improved equally regarding lin-guistic skills, communicative ability (patients' and caregivers' perspective) and quality of life; within-group comparisons showed improve-ment in more AAT subtests and WHOQoL sub-domains in VR group compared to non-VR-group; no follow-up
					CG: n = 18	CG: Conversational group treatment without VR, 3 participants & 1 SLP/group session			

[1] The information about the treatment dose was not entirely clear in the publication by Giachero et al. (2020): It was reported that over a period of 24 weeks, treatment was performed twice a week and that each treatment lasted two hours, which would suggest a total dose of 96 hours. However, the total dose was specified as 48 hours in the text. Since the corresponding author did not reply to a query on this matter, we assume that each session did not take two but one hour, summing up to the reported 48 hours.

ID	Study	Design	Etiology	Syndrome/ severity	Sample size	Intervention	Length/fre-quency	Outcome measures	Main result
89	Kristensson et al. (2022)	RCT	Stroke	Mild to se-vere apha-sia	IG: n = 9	IG: Naming group treat-ment based on modified semantic feature analysis (SFA), 3-5 participants & 1 SLP/group session	20 sessions of 2 h each, 3 times per week, 40 h in total	Object and Action Nam-ing Battery (OANB), Bos-ton Naming Test (BNT), Amsterdam–Nijmegen Everyday Language Test (ANELT), General Short Form of the Communica-tive Participation Item Bank (CPIB), Stroke and Aphasia Quality of Life Scale-39 (SAQOL-39), Carer Communication Outcomes after Stroke (COAST) scale	Improved naming ability for trained items in both groups; no changes in communication or quality of life; effect not maintained at 10-weeks follow-up
					CG: n = 8	CG: Group exercises on au-ditory comprehension, cop-ying text, and reading, 3-5 participants & 1 SLP/group session			
82	Küst et al. (2009)	RCT	Stroke	Residual to severe aphasia	IG: n = 13	IG: CIAT, 2-3 participants & 2 SLP/group session	IG: 3 h/day, 5 days/week, for 6 weeks, 90 h in total	Aachen Aphasia Test (AAT), Aphasia Check List (ACL), Communicative Ef-fectiveness Index (CETI)	Both groups improved in their linguistic and communicative abilities; greater increase in word generation after usual care compared to CIAT; better performance in repetition and au-tomatic speech tasks after CIAT compared to usual care
					CG: n = 15	CG: Usual care in individual setting	CG: 45 min/day, 5 days/week, for 6 weeks, 22.5 h in total		
67	Marshall et al. (2016)	Crosso-ver RCT	Stroke	Mild to moderate aphasia	IG: n = 10	IG: Immediate conversa-tional group treatment us-ing VR-platform EVA Park, 5 participants and 1 SLP or experienced volun-teer/training cohort	25 sessions of 1 h each, 5 days/week, for 5 weeks, 25 h in total	Communication Activities of Daily Living (CADL-2) test; verbal fluency (se-mantic); conversation analysis based on Profile of Word Errors and Re-trieval in Speech	Functional communication improved during treatment but not during waiting period; effect was maintained at 13-week follow-up; no inter-vention-associated change in communication confidence or feelings of social isolation

ID	Study	Design	Etiology	Syndrome/ severity	Sample size	Intervention	Length/fre- quency	Outcome measures	Main result
					CG: n = 10	CG: Deferred treatment; no intervention while awaiting treatment		(POWERS); analysis of narrative production (re-telling the story of Cinder-ella) using methods form the Quantitative Produc-tion Analysis; The Com-munication Confidence Rating Scale for Aphasia (CCRSA); Friendship Scale	
70	Marshall et al. (2018)	Crosso-ver CCT	Stroke	Mild to se-vere apha-sia	IG: n = 5	IG: Immediate guided group Mindfulness Meditation (MM), 2-3 participants & 1 SLP/group session	IG: 5 sessions, duration in-creasing from 10 to 30 min on 5 consecutive days, 1.6 – 2 h in total	Western Aphasia Bat-tery—R (WAB-R), Con-ners' Continuous Perfor-mance Test—II (CPT - II), Centre for Research on Safe Driving-Attention Network Test (CRSD-ANT), Helm-Estabrooks and Nicholas Narrative Story Cards, Revised Token Test - 5, Aphasia Diagnostic Profiles (ADP); telemetric heart rate monitor (HRM); Salivette for sali-vary samples	No significant gains in attention or language measures; no group effect for physiological measures; MM group: positive correlation be-tween change in heart rate and both word productivity and error frequency in storytelling task, negative correlation between change in heart rate variability and word productivity
					CG: n = 3	CG: Deferred treatment; group mind-wandering dur-ing waiting period, 3 partici-pants & 1 SLP/group session	CG: Before in-tervention 5 sessions of wait-list program on 5 consecutive days, 1.6 – 2 h in total		
83	Meinzer et al. (2005)	CCT	Stroke	Mild to se-vere apha-sia	IG: n = 12	IG: CIAT; 2-3 participants & 2 SLPs/group session	3 h/day over a 2-week period, 30 h in total	Aachen Aphasia Test (AAT), Communicative Ef-fectiveness Index (CETI), Communicative Activity Log (CAL) (patient and rel-atives' version)	Both groups improved in language functions and ratings of communicative effectiveness and amount of everyday communication (rela-tives and patient perspective); effect was maintained at 6-months follow-up; increase in communicative measures was higher in CIAT-plus group
					CG: n = 15	CG: CIATplus, 2-3 partici-pants & 2 SLPs/group ses-sion			
66	Mohr et al. (2017)	Crosso-ver RCT	Stroke, TBI, viral enceph-alopa-thy	Mild to se-vere nonflu-ent aphasia	IG: n = 8	IG: Intensive language ac-tion therapy (ILAT) followed by intensive naming ther-apy (INT), 3 participants & 1 SLP/group session	3.5 h/day for 6 consecutive working days, 21 h in total	Aachen Aphasia Test (AAT): subscales token test, repetition, naming, and auditory comprehen-sion, shortened 20-items	Better language performance after ILAT than after INT; reduction of depressive symptoms after ILAT, but not after INT

ID	Study	Design	Etiology	Syndrome/ severity	Sample size	Intervention	Length/fre-quency	Outcome measures	Main result
					CG: n = 9	CG: INT followed by ILAT, 3 participants & 1 SLP/group session		version of the Beck depression inventory (BDI-5)	
84	Nenert et al. (2022)	RCT	Stroke	Mild to severe aphasia	IG: n = 11	IG: CIAT, 3-4 participants & 1 SLP/group session	4 h/day for 10 days, 40 h in total	Peabody Picture Vocabulary (PPVT); Boston Naming Test (BNT); Controlled Oral Word Association Test (COWAT); Semantic Fluency Test (SFT), Mini-Communicative Activities Log (Mini-CAL), data collection during fMRI tasks: semantic decision/tone decision and covert verb generation	Naming improved in treated and untreated patient group until 3-months follow-up; no change in other scores; enhanced naming performance was associated with activation patterns predominantly in the right, but also in the left hemisphere (temporal gyrus, postcentral gyrus, precentral gyrus, thalamus, left middle and superior frontal gyri)
					CG: n = 46 (untreated n = 8; healthy n = 38)	CG: no intervention			
87	Rose et al. (2022)	RCT	Stroke	Mild to severe aphasia	IG: n = 146 (CIATplus n = 71; Multi-Modal Aphasia Therapy - M-MAT n = 75)	IG: CIATplus or M-MAT, 3 participants & 1 SLP/group session	IG: 3 h/day, 5 days/week for two weeks, 30 h in total	Western Aphasia Battery—R Aphasia Quotient (WAB-R AQ); COMPARE naming battery; correct information units (CIUs)/ min; Communication Effectiveness Index (CETI); Scenario Test; Stroke and Aphasia Quality of Life Scale-39g (SAQOL-39g)	CIATplus and M-MAT both improved word retrieval, functional communication, and communication-related quality of life (CIATplus: (MD 0.20; 95% CI 0.01 to 0.39; p = 0.039; M-MAT: MD 0.43; 95% CI 0.24 to 0.62; p = 0.0001), with M-MAT being superior for communication-related quality of life (MD 0.24; 95% CI 0.05 to 0.42; p = 0.008) and CIATplus superior for word retrieval; effect for word retrieval maintained at 12 weeks follow-up
					CG: n = 70	CG: Usual care (individual, computerized, or social/support group sessions)	CG: no treatment or < 1 h/week, < 2 h in total		
86	Stahl et al. (2018)	RCT	Stroke	Aphasia with heterogenous symptom severity	IG: n = 15	IG: 2 training intervals of ILAT in highly intense dose after initial waiting period, 3 participants & 1 SLP/group session	IG: 4h/day, 3 days/week for 4 weeks, 48 h in total	Aachen Aphasia Test (AAT): subscales token test, repetition, naming and comprehension; Action Communication Test (ACT)	Compared to waiting period, linguistic performance improved in both groups independent of intensity over entire therapy phase; during second training interval, progress in communicative-pragmatic skills only continued in the moderately intense group
					CG: n = 15	CG: 2 training intervals of ILAT in moderately intense dose after initial waiting period, 3 participants & 1 SLP/group session	CG: 2h/day, 3 days/week for 4 weeks, 24 h in total		

ID	Study	Design	Etiology	Syndrome/ severity	Sample size	Intervention	Length/frequency	Outcome measures	Main result
85	Vuksanović et al. (2018)	Crossover RCT	Stroke	Mild to severe nonfluent aphasia	IG: n = 10	IG: 2 successive trainings blocks with multimodal stimulation aphasia therapy (SAT) first, followed by CIAT (C1S2), 2 participants & 1 SLP/group session	For each of the two training blocks 1h/day, 5 days/week for 4 weeks, 20 h per block, 40 h in total	Boston Naming Test (BNT; Cookie Theft Picture description task: total number of sentences (NS), syntactic diversity (SD), total number of information carrying words (NICW)	Improvement of expressive language skills in both groups; effects maintained at 1-month follow-up; CIAT was associated with greater gains in naming compared to SAT; the group with CIAT first (C1S2) improved more in naming ability and number of sentences produced than the group with SAT first (S1C2)
					CG: n = 10	CG: 2 successive trainings blocks with CIAT first, followed by SAT (S1C2), 2 participants & 1 SLP/group session			
88	Wertz et al. (1981)	RCT	Stroke	Aphasia (percentile 16-74 on Porch Index of Communicative Ability - PICA)	IG: n = 35 (fractioned to cohorts with 11, 22, and 33 weeks of treatment)	IG: Usual care (multi-modal stimulus-response type treatment in individual setting, supplemented by machine-assisted treatment and speech and language drill)	IG: 4 h/week of individual treatment (plus 4 h/week of supplemental treatment for 44 weeks), 176 h of individual treatment in total	Porch Index of Communicative Ability (PICA), Token Test, Word Fluency Measure; a motor speech evaluation adapted from Wertz and Rosenbek (1971); Coloured Progressive Matrices (CPM); a Conversational Rating developed for the study, an Informant's Rating of functional language use adapted from Sarno (1969)	Both treatment groups improved on all measures; most improvement occurred during the first 11-week treatment period; longer treatment was associated with better outcomes; individual treatment resulted in more improvement on all measures in all cohorts except for better performances after group treatment regarding gesture production (PICA), word fluency and nonverbal intelligence (CPM); no follow-up
					CG: n = 32 (fractioned to cohorts with 11, 22, and 33 weeks of treatment)	CG: Conversational group treatment, supplemented by recreational group activities, 3-7 participants & 1 SLP/group session	CG: 4 h/week of group treatment (plus 4 h/week of supplemental activities for 44 weeks), 176 h of group treatment in total		

Table 3: Summary of systematic reviews PT/SLT

ID	Discipline/study		Design	Etiology	Syndrome/ severity	Number of included studies	Sample size	Intervention	Length/frequency	Main outcome measures	Main result
74	PT	English et al. (2017)	Systematic review with meta-analysis	Stroke	Mild to moderate	N=17	IG: n = 423<br>CG: n = 415	CCT vs. 'other-intervention'	Length: 2 weeks to 1 year; Sessions: 1-5 times/week Sum of sessions: 7 to 78 per Intervention; length of each session: 30min-120min	Mobility/ Walking Capacity	For their primary outcome measure of gait capacity, they found CCT to be superior to other interventions for improving the distance walked on the 6mWT. Meta-analysis demonstrated that overall CCT was superior to the comparison intervention (MD 60.86, 95% CI 44.55 to 77.17; I 2 = 27%) GRADE: moderate
							IG: n = 131<br>CG: n =138	CCT +education vs. no intervention			
							IG: n = 48<br>CG: n =	CCT vs. different CCT			
80	SLT	Zhang et al. (2017)	Systematic review with meta-analysis	Stroke	Mild to severe aphasia	n = 8	IG: n = 64<br>CG: n =37	CIAT vs. usual/no care	6 days - 5 weeks; 45 min - 4 h/day	Aachen Aphasia Test (AAT), Boston Naming Test (BNT)	Meta-analysis (not all studies included) revealed no significant difference between CIAT and other intensive therapy programs regarding language performance (AAT: 2 Studies with 109 participants; BNT: 2 studies with 33 participants); limited evidence to support its superiority over other therapy approaches
							IG: n = 79<br>CG: n = 74	CIAT vs. intensive unconstraint therapy			
							IG: n = 9<br>CG: n = 9	ILAT vs. intensive naming therapy			

Table 4: Summary of PT primary studies

ID	Study	Design	Etiology	Syndrome/ severity	Sample size	Intervention	Length/frequency	Outcome measures	Main result
75	Dickstein et al. (2014)	Crossover CCT	Stroke	Mild	IG: n = 8	group-based imagery training with kinesthetic focus + 2x daily 5min home exercise of the group content	≈30min Sessions/ 10 min motor imagery practice the rest of the time are descripted as Warming up, Cooling down, Feedback and Demonstration; 2x per week for 5 weeks	10m walking test; vertical forces during gait; Tinetti gait test; ABC-Scale; Box and Blocks Test; VAS; Kinesthetic and Visual Imagery questionnaire (KVIQ)	No differences between the effects of the experimental and the control treatments for any of the tested outcomes
					CG: n = 8	group-based imagery training with visual focus + 2x daily 5min home exercise of the group content			
63/ 62	Doussoulin et al. (2017) and Doussoulin et al. (2018)[2]	RCT/ Randomized clinical trail	Stroke	Mild to moderate	IG: n = 24	repetitive, task-oriented training, induction in the use of the affected upper extremity by motor constraint less-affected side (CIMT) and behavioral technics in a group modality	3h daily for 10 days	Functional Independence Measure scale (FIM)	The motor subscale of the FIM presents differences in favor of the individual modality ($p<0.001$; $\eta^2_p = 0.70$). The cognitive subscale showed differences in favor of the collective modality ($p<0.001$; $\eta^2_p = 0.73$)., same as the total score ($p<0.001$; $\eta^2_p = 0.79$).
					CG: n = 12	repetitive, task-oriented training, induction in the use of the affected upper extremity by motor constraint less-affected side (CIMT) and behavioral technics in a individual modality		Motor Activity Log30 (MAL-30); Action Research Arm Test (ARAT)	The MAL-30 scores show a significant difference between the groups in favor for the group modality ($p<0.001$; $\eta^2_p = 0.57$). The mean scores of ARAT increased from the pretest to the post-test by 4.95 points for the CIMT group and by 4.94 points for the individual CIMT group. An ANCOVA indicated a significant difference between the treatment conditions favoring the

[2] The two publications are combined because only the outcomes differ and to avoid duplication of study sample in our synthesis.

ID	Study	Design	Etiology	Syndrome/ severity	Sample size	Intervention	Length/frequency	Outcome measures	Main result
									group therapy, after controlling for the pretest scores ($p = 0.016$, $\eta^2_p = 0.22$).
71	English et al. (2007)	CCT	Stroke	Mild to severe	IG: n = 38	Circuit class therapy: Training of core activities that addressed the patients' key impairments and functional limitations	two 90-minute treatment sessions a day, 5 days a week for more than 4 weeks	Primary outcome: five-meter walk test (5MWT), two-minute walk test (2MWT), Berg Balance Scale (BBS) secondary outcome: Motor Assessment Scale (MAS) for stroke, Iowa Level of Assistance Scale (ILAS)	Subjects receiving either circuit class therapy or individual therapy demonstrated a similar degree of recovery on objective measures of mobility and upper-limb function for person receiving inpatient rehabilitation after stroke. However, circuit class therapy was associated with a significantly greater degree of independence in walking at discharge from rehabilitation and significantly higher patient satisfaction with the amount of therapy received. But in the 2MWT the individual modality was significantly superior to the circuit class therapy. Furthermore, the study demonstrated the feasibility and safety of circuit class therapy as an alternative sole model of PT service delivery for person receiving inpatient rehabilitation after stroke.
					CG: n = 40	Standard individual care based on no particular treatment philosophy	60 minutes a day, 5 days a week for more than 4 weeks		
65	Kleffelgaard et al. (2019)	RCT	TBI	moderate	IG: n = 33	multidisciplinary outpatient rehabilitation + group-based vertigo rehabilitation (CCT with different balance tasks, exercises for lower limp strength and exercises for gaze stability) + home-based exercise program	2h sessions 2x per week over 8 weeks; 16 sessions in total	Dizziness Handicap Inventory (Norwegian); High-level mobility assessment tool for traumatic brain injury (Norwegian); Vertigo Symptom Scale-Short Form (subscale vertigo-balance and autonomic-anxiety); Rivermead Post-concussion Symptoms Questionnaire (subscale physical and	At the first post-intervention follow-up, a statistically significant between-group mean difference was found in the respective primary and secondary outcome measures in favor of the intervention group. At the second post-intervention follow-up, the between-group differences were no longer statistically significant. The intervention group maintained their level of

ID	Study	Design	Etiology	Syndrome/ severity	Sample size	Intervention	Length/frequency	Outcome measures	Main result
						(walking, biking, swimming)		psychological); Hospital Anxiety and Depression Scale (subscale anxiety and depression; Balance Error Scoring System	improvement, while the control group improved over time in dizziness-related disability and mobility problems. No significant differences between the groups were found in any of the other outcomes at the two post-intervention follow-ups.
					CG: n = 32	multidisciplinary outpatient rehabilitation (+ reposition maneuvers, if positioning test was positive)			
77	Malagoni et al. (2016)	RCT	stroke	moderate	IG: n = 6	Exercise therapy in group modality consisting of 20 min of endurance training (level walking, stair climbing) and 40 min task-orientated training targeting balance, strength, and flexibility.	1h sessions 3 times/week for 10 weeks	6mWT; TUG; Stair climb test (SCT); SF-36; client satisfaction Questionnaire (csQ-8); calculation of adherence score	No statistically significant intergroup differences were found.
					CG: n = 6	Home-based walking exercise program (Test In-Train Out program) using a metronome. The program is designed progressive regarding walking intensity, based on 2-minute walking test result.	2x 10-minute sessions/day at 6 days/week for 10 weeks		
72	Martins et al. (2020)	RCT	stroke	Mild to moderate	IG: n = 18	Circuit class therapy including activities of reaching, grasping, manipulation of different objects, writing, sit-to-stand, step and heel raise activities and	1h sessions 3x/week for 12 weeks (36 sessions in total)	primary outcomes: PA monitor (SenseWear®), Human Activity Profile (HAP), 10 meter walking test, Test d'Evaluation des Membres Superieurs de Personnes Agees (TEMPA)	There were not found any interactions or between-group effects for any of the outcomes, except for quality of life, which improved for the experimental group after intervention and 16 week follow-up (p = 0.02).

ID	Study	Design	Etiology	Syndrome/ severity	Sample size	Intervention	Length/frequency	Outcome measures	Main result
						walking. 50/50 upper and lower limb		secondary outcomes: portable dynamometry, 6minWT, SSQOL	
					CG: n = 18	Stretching + Education/ health information + memory exercise			
78	Noh et al. (2008)	RCT	Stroke	Mild to moderate	IG: n = 13	group-based aquatic therapy targeting balance (based on the Halliwick and Ai Chi methods	1h sessions 3x/week for 8 weeks	Chedoke–McMaster Stroke Assessment scores; Berg Balance Scale; mtd-Balance; Modified Motor Assessment Scale; Biodex Medical Systems	The improvement in the Berg Balance Scale score was greater in the aquatic therapy group than in the conventional therapy group (p = 0.032; effect size: 1.03). Among the weightbearing parameters, improvements in forward (p = 0.044; effect size: 1.14) and backward weight-bearing abilities (p = 0.031; effect size: 0.72) were greater in the aquatic therapy group than in the conventional therapy group. The change in the torque of the knee flexor on the affected side was greater in the aquatic therapy group than in the conventional therapy group (p = 0.037; effect size: 1.13). There was no significant difference in trunk muscle strength between the groups.
					CG: n = 12	land-based group-based exercise therapy (lower and upper limb) and gait training			
12	Renner et al. (2016)	RCT	stroke	moderate	IG: n = 34	Circuit class therapy intended to improve tasks relating to walking competency	90min Sessions 5x/ week for 6 weeks	primary outcome: Stroke Impact Scale (version 3.0) mobility scale secondary outcome: all other domains of the Stroke Impact Scale (version 3.0): the Rivermead mobility index; the Falls Efficacy scale (international version); the Hospital Anxiety and Depression scale and the Fatigue	The Motricity index, 6 minute walk test, timed up and go test, and modified stairs climb test showed significantly better results in favor of the patients assigned to the group therapy task training; The linear mixed-models analysis (with time and group as fixed effects, and with subjects as a random effect) demonstrated a significant between group effect favoring the group therapy task
					CG: n = 39	Individual exercise therapy aimed to improve balance, physical condition and walking competency			

ID	Study	Design	Etiology	Syndrome/ severity	Sample size	Intervention	Length/frequency	Outcome measures	Main result
								Severity scale; Motricity index; functional ambulation categories; six minute walk test; ten meter comfortable walking speed test; timed balance test; timed up and go; chair rise test and modified stairs climb test; letter cancellation task.	training for the ten meter comfortable walking speed test (P<0.0001) and for the six minute walk test (P<0.0001) after correcting for multiple comparisons.
79	Stein et al. (2014)	RCT	Stroke	Mild to moderate	IG[3]: n = 12	group therapy with the aim to control for the effects of interacting with a physical therapist by providing an equivalent number of formal therapy sessions while providing minimal gait training.	1h Sessions 3x/week for 6 weeks (18 sessions in total)	TUG; 10m-walk test; 6minWT; 5times Sit-to-stand test; Berg Balance scale; California functional evaluation 40; Emory Functional Ambulation Profile (EFAP);	No significant differences were found between the two groups for the primary outcome measure at week 6. Results for the Berg Balance scale favored the robotic treatment group, while EFAP results favored the control exercise group. Other secondary measures showed no differences between the groups
					CG: n = 12	individual gait training with robotic device			
73	Stibrant Sunnerhagen (2007)	CCT	Stroke	Mild to moderate	IG: n = 21	Circuit class therapy: progressive resistance strength training for the lower limb with the patients completing practice at a series of workstations	45min Sessions 3x per week for 8 weeks	Dynamometry; electrical bicycle ergometer (CCE 2000, Medical Graphic Corp, St Paul, Minn); 30m gait test (normal and maximum speed); Instrumental Activity Measure; Physical Activity Scale for the Elderly (PASE)	No significant changes were observed after the training period in self-selected or maximal walking speed, personal or instrumental ADL capacity, or activity level (PASE). The paretic leg showed a difference in favor of the IG in Dynamometry at 60-degree knee angle during extension ($p < 0.05$), during 60 degrees/s extension ($p < 0.05$), and flexion ($p < 0.05$).
					CG: n = 9	No therapy			

[3] IG was CG group in original study

ID	Study	Design	Etiology	Syndrome/ severity	Sample size	Intervention	Length/frequency	Outcome measures	Main result
76	Thieme et al. (2012)	RCT	Stroke	Severe (motoric)	IG: n = 21	Group mirror therapy	30min Sessions daily for 5 weeks (in total 20 sessions)	primary outcome: Fugl-Meyer Assessment-arm section (FMA); Action Research Arm test (ARAT) secondary outcome: Barthel-Index; Stroke Impact Scale; Fugl-Meyer test-arm section; modified Ashworth scale; star cancellation test	There were no significant differences between the groups in the Fugl-Meyer test, Barthel Index or ARAT. Significant group difference in the mod. Ashworth scale between individual mirror therapy and the mirror therapy group intervention, indicating higher scores for individual mirror therapy after the intervention ($p < 0.05$); They found a significant group interaction for the Star Cancellation Test score over time ($F = 7.5$, $P = 0.009$). Post-hoc analysis revealed a significant group difference between individual mirror therapy and the control group ($P < 0.01$)
					CG1: n = 18	Individual mirror therapy			
					CG2: n = 21	Sham group mirror therapy			
